# Crystal structure and Hirshfeld surface analysis of 5-(3,5-di-*tert*-butyl-4-hy­droxy­phen­yl)-3-phenyl-4,5-di­hydro-1*H*-pyrazole-1-carboxamide

**DOI:** 10.1107/S205698901901243X

**Published:** 2019-09-12

**Authors:** Ayten R. Asgarova

**Affiliations:** a Organic Chemistry Department, Baku State University, Z. Khalilov str. 23, Az, 1148 Baku, Azerbaijan

**Keywords:** crystal structure, pyrazole, carboxamide, 3,5-di-*tert*-butyl-4-hy­droxy­phen­yl, N—H⋯O hydrogen bonds, inversion dimer

## Abstract

In the title compound, C_24_H_31_N_3_O_2_, the mean plane of the central pyrazole ring [r.m.s. deviation = 0.095 Å] makes dihedral angles of 11.93 (9) and 84.53 (8)°, respectively, with the phenyl and benzene rings. There is a short intra­molecular N—H⋯N contact, which generates an *S*(5) ring motif. In the crystal, pairs of N—H⋯O hydrogen bonds link inversion-related mol­ecules into dimers, generating an *R*_2_^2^(8) ring motif. The Hirshfeld surface analysis indicates that the most significant contribution involves H⋯H contacts of 68.6%

## Abstract 2

In the title compound, the mean plane of the central pyrazole ring [r.m.s. deviation = 0.095 Å] makes dihedral angles of 11.93 (9) and 84.53 (8)°, respectively, with the phenyl and benzene rings. In the crystal, pairs of N—H⋯O hydrogen bonds link inversion-related mol­ecules into dimers, generating an 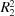
(8) ring motif.

## Chemical context   

Compounds containing the pyrazole ring system, considered to be a pharmacologically important active scaffold, possess diverse biological activities such as anti­microbial, anti-inflammatory, analgesic, anti­convulsant, anti­cancer, anthelmintic, anti­oxidant and herbicidal (Ansari *et al.*, 2017 ▸; Karrouchi *et al.*, 2018 ▸; Mamedov *et al.*, 2017 ▸). Such compounds have been the subject of NMR investigations of hydrogen bonding and keto–enol tautomerism in solution (Mamedov *et al.*, 2013 ▸, 2015 ▸). The structural properties of a series of compounds derived from 2,6-di-*tert*-butyl­phenol have been characterized in the solid state (Asgarova *et al.*, 2011*a* ▸,*b* ▸, 2019 ▸; Khalilov *et al.*, 2018*a* ▸,*b* ▸). Non-covalent bond donor/acceptor properties of pyrazoles or related N-compounds are crucial in the organization of supra­molecular architectures in the solid state and hence their catalytic activity, solubility, *etc*. (Ma *et al.*, 2017 ▸; Maharramov *et al.*, 2010 ▸; Mahmoudi *et al.*, 2016 ▸, 2017*a* ▸,*b* ▸, 2018*a* ▸,*b* ▸,*c* ▸; Mahmudov *et al.*, 2014 ▸, 2019 ▸; Shikhaliyev *et al.*, 2018 ▸). As part of a further study of this class of compounds, the crystal structure and Hirshfeld surface analysis of the title compound, 5-(3,5-di-*tert*-butyl-4-hy­droxy­phen­yl)-3-phenyl-4,5-di­hydro-1*H*-pyrazole-1-carboxamide, are reported on herein.
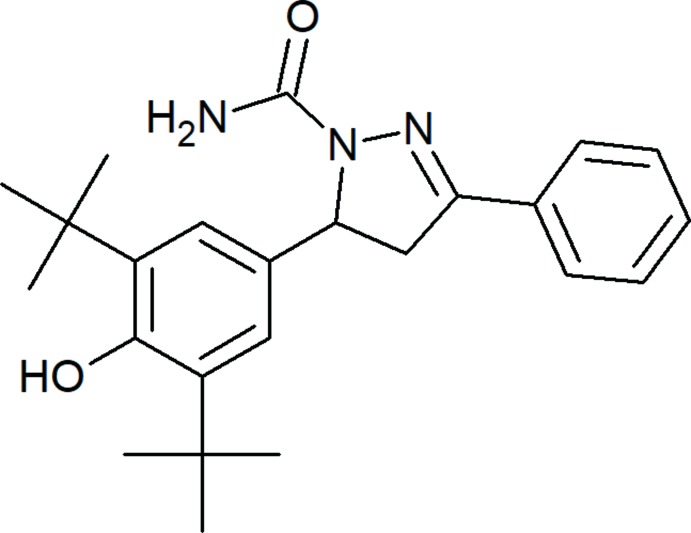


## Structural commentary   

**Figure 1:**
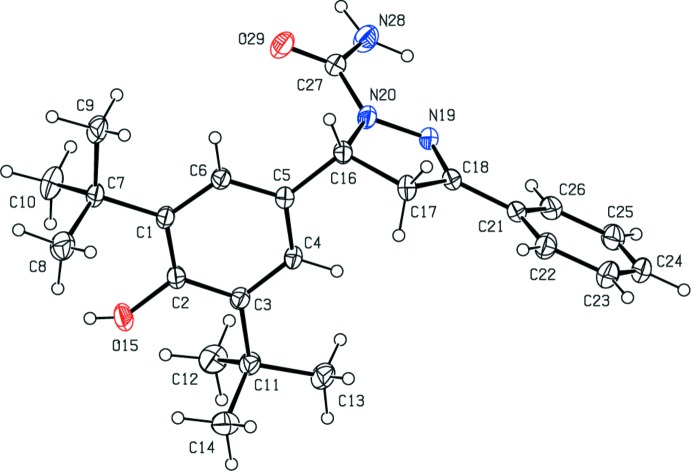
The mol­ecular structure of the title compound, with the atom labelling. Displacement ellipsoids are drawn at the 30% probability level.

As shown in Fig. 1 ▸, the title mol­ecule contains three rings, pyrazole ring *A* (N19/N20/C16–C18; twisted conformation on bond C16-C17), phenyl ring *B* (C21–C26) and benzene ring *C* (C1–C6), with rings *B* and *C* being inclined to the mean plane of the central pyrazole ring *A* [r.m.s deviation = 0.095 Å] by 11.93 (9) and 84.53 (8)°, respectively. In the >NC(=O)NH_2_ group, atoms N20, C27, O29 and N28 are coplanar, with N19—N20—C27—N28 and N19—N20—C27—O29 torsion angles of 4.0 (2) and −176.1 (1)°. All bond lengths and angles are comparable with those found for closely related structures, for example, methyl 3-(3,5-di-*tert*-butyl-4-hy­droxy­phen­yl)pro­pionate (Li *et al.*, 2014 ▸), 2,6-di-*tert*-butyl-4-methyl­phenol (Iimura *et al.*, 1983 ▸), 2,6-di-*tert*-butyl-4-(3-chloro-2-hy­droxy­prop­yl)phenol (Asgarova *et al.*, 2011*a* ▸) and 4-[3-(benzyl­amino)-2-hy­droxy­prop­yl]-2,6-di-*tert*-butyl­phenol (Asgarova *et al.*, 2011*b* ▸). In the mol­ecule, there is an N—H⋯N short contact, which generates an *S*(5) ring motif (Table 1 ▸).

## Supra­molecular features and Hirshfeld surface analysis   

**Figure 2:**
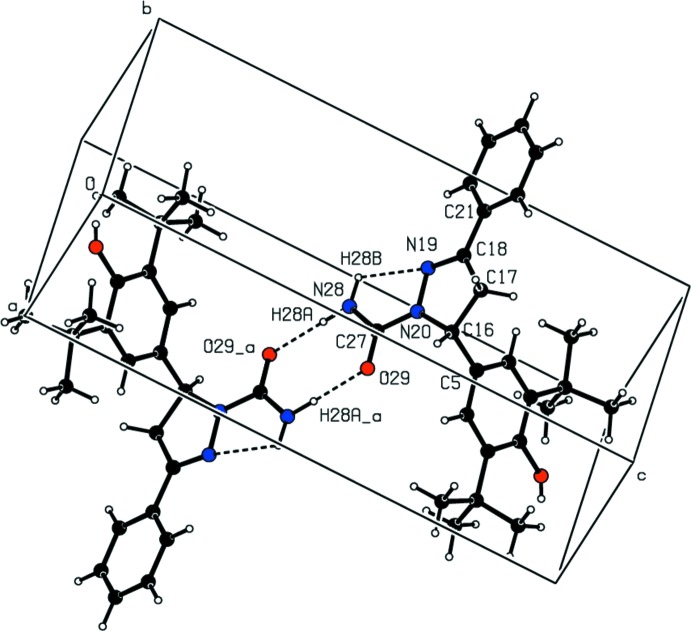
A view of the dimeric mol­ecular bonding formed by N—H⋯O hydrogen bonds and N—H⋯N short contacts (dashed lines), with an *S*(5)
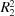
(8)*S*(5) motif [symmetry code: (a) −*x* + 2, −*y* + 1, −*z* + 1].

In the crystal, pairs of N—H⋯O hydrogen bonds link inversion-related mol­ecules into dimers, generating an 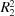
(8) ring motif (Table 1 ▸; Fig. 2 ▸). No C—H⋯π or π-π inter­actions are present in the crystal structure (*PLATON*; Spek, 2009 ▸).

**Figure 3:**
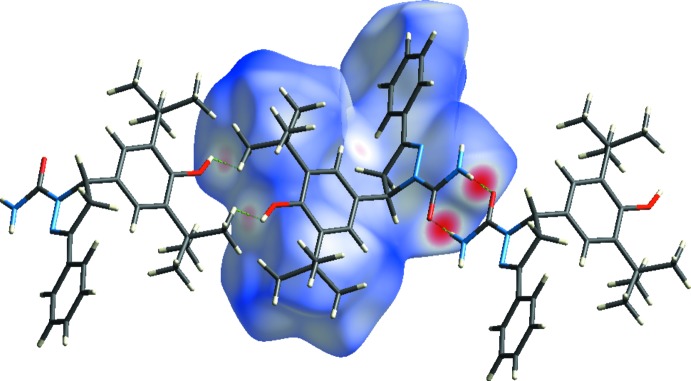
View of the three-dimensional Hirshfeld surface of the title compound mapped over *d*_norm_, in the range −0.5426 to 1.7721 au.

**Figure 4:**
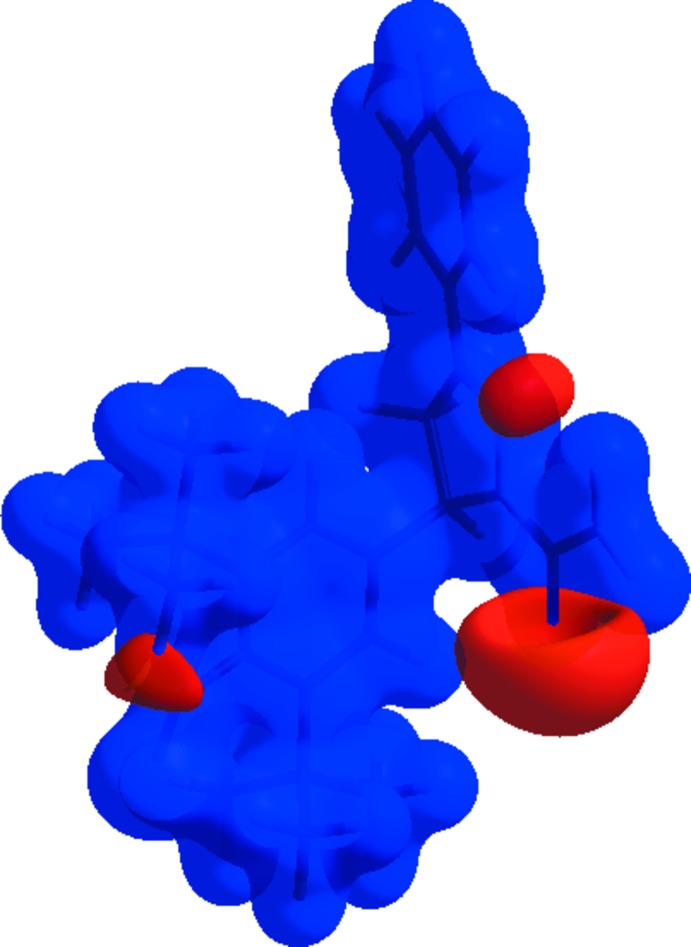
View of the three-dimensional Hirshfeld surface of the title compound mapped over the electrostatic potential energy in the range −0.0500 to 0.0500 a.u. using the STO-3 G basis set at the Hartree–Fock level of theory. Hydrogen-bond donors and acceptors are shown as blue and red regions around the atoms corresponding to positive and negative potentials, respectively.

The Hirshfeld surface analysis (Spackman & Jayatilaka, 2009 ▸) was generated by *CrystalExplorer*17 (Turner *et al.*, 2017 ▸) and comprises *d*_norm_ surface plots and two-dimensional fingerprint plots (Spackman & McKinnon, 2002 ▸). A *d*_norm_ surface plot of the title compound mapped using a standard surface resolution with a fixed colour scale of −0.5426 (red) to 1.7721 a.u. (blue) is shown in Fig. 3 ▸. The dark-red spots on the *d*_norm_ surface arise as a result of the N—H⋯O hydrogen bonds (Table 1 ▸), while the other weaker inter­molecular inter­actions appear as light-red spots. The bright-red spots indicate their roles as the respective donors and/or acceptors; they also appear as blue and red regions corresponding to positive and negative potentials on the Hirshfeld surfaces mapped over electrostatic potential (Spackman *et al.*, 2008 ▸; Jayatilaka *et al.*, 2005 ▸), as shown in Fig. 4 ▸.

**Figure 5:**
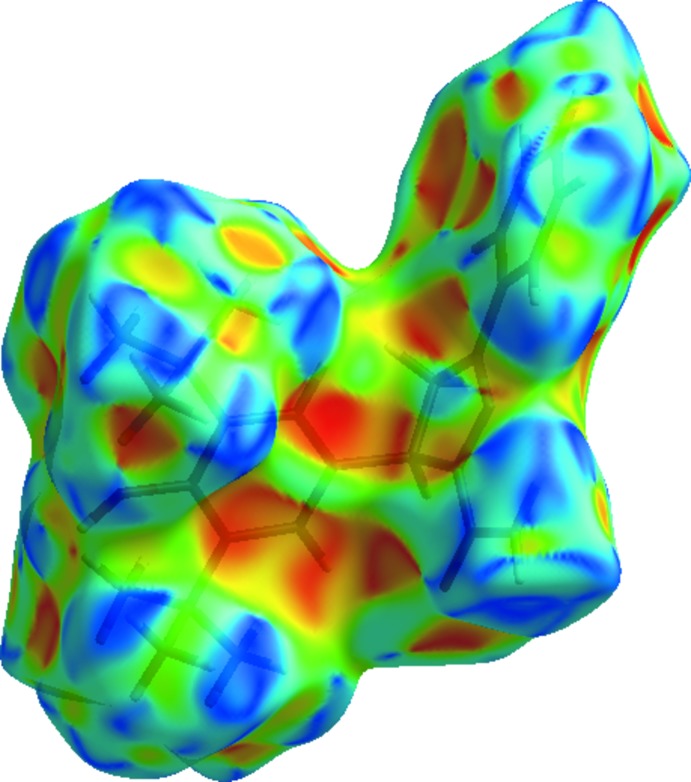
Hirshfeld surface of the title compound mapped over the shape-index.

**Figure 6:**
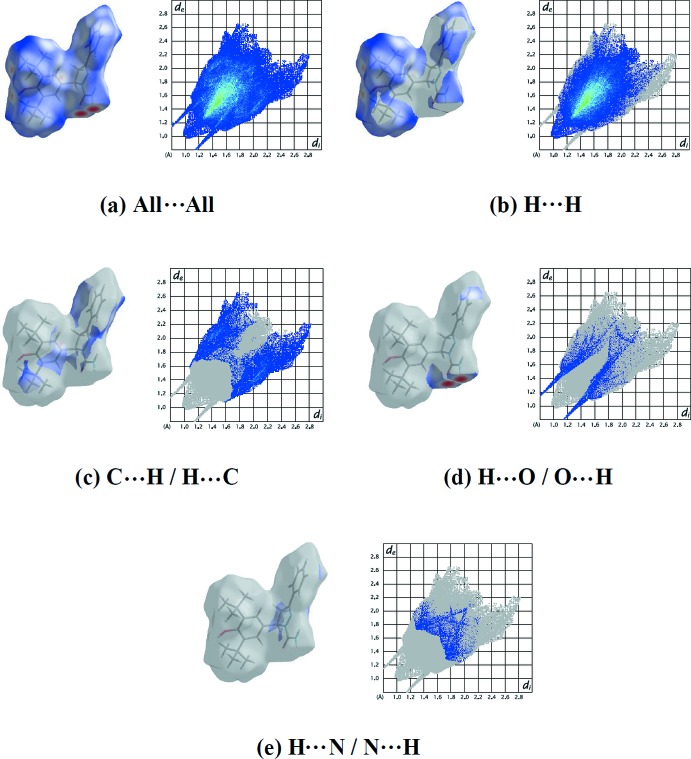
The Hirshfeld surface representations and the full two-dimensional fingerprint plots for the title compound, showing (*a*) all contacts, and delineated into (*b*) H⋯H, (*c*) C⋯H/H⋯C, (*d*) H⋯O/O⋯H and (*e*) H⋯N/N⋯H contacts. The *d*_i_ and *d*_e_ values are the closest inter­nal and external distances (in Å) from given points on the Hirshfeld surface.

The shape-index of the Hirshfeld surface is a tool to visualize π–π stacking inter­actions by the presence of adjacent red and blue triangles; if there are no adjacent red and/or blue triangles then there are no π–π inter­actions. Fig. 5 ▸ clearly indicates that there are no π–π inter­actions present in the the crystal of the title compound, as also indicated by the analysis of the crystal structure using *PLATON* (Spek, 2009 ▸). Fig. 6 ▸ *a* shows the two-dimensional fingerprint of the sum of the contacts contributing to the Hirshfeld surface represented in normal mode. These represent both the overall two-dimensional fingerprint plot and those delineated into H⋯H (68.6%), C⋯H/H⋯C (18.3%), H⋯O/O⋯H (7.1%) and H⋯N/N⋯H (4.1%) contacts (Fig. 6 ▸ *b*–*e*). The most significant contribution to the Hirshfeld surface is from H⋯H contacts (68.6%; Fig. 5 ▸ *b*).

The large number of H⋯H, C⋯H/H⋯C, H⋯O/O⋯H and H⋯N/N⋯H contacts suggest that van der Waals inter­actions and hydrogen bonding play the major roles in the crystal packing (Hathwar *et al.*, 2015 ▸).

## Synthesis and crystallization   

To a solution of of 3-(3,5-di-*tert*-butyl-4-hy­droxy­phen­yl)-1-phenyl­prop-2-en-1-one (1.2 mmol) in 10 ml ethanol was added semicarbazide hydro­chloride (1.26 mmol). The mixture was refluxed for 3 h and then cooled to room temperature. The title compound, that precipitated as colourless single crystals, was collected by filtration and washed with an ethanol–water (1:1) mixture (yield 56%, m.p. 525 K). ^1^H NMR (300 MHz, DMSO-*d*_6_) : 1.38 (*s*, 18H, 6CH_3_); 3.05 (*dd*, 1H, CH_2_, ^3^ *J*_H-H_ = 4.8, ^2^ *J*_H-H_ =17.7,); 3.75 (*dd*, 1H, CH_2_, ^3^ *J*_H-H_ = 12, ^2^ *J*_H-H_ = 17.7), 5.35 (*dd*, 1H, CH_2_, ^3^ *J*_H-H_ = 4.8, ^2^ *J*_H-H_ = 11.7); 6.51 (*s*, 2H, NH_2_); 6.87 (*s*, 1H, OH_ar_); 6.96 (*s*, 2H, 2Ar-H); 7.41–7.83 (*m*, 5H, 5Ar-H). ^13^C NMR (75 MHz, DMSO-*d*_6_): 30.79 (6CH_3_), 34.94 (2C_*tert*_), 43.00 (CH_2_), 60.32 (CH), 121.79 (2CH_ar_), 126.90 (2CH_ar_), 129.39 (2CH_ar_), 130.02 (CH_ar_), 132.18 (C_ar_), 134.96 (C_ar_), 139.67 (C_ar_), 151.13(N=C_*tert*_), 153.23 (O—C_ar_) 155.56 (NC=O).

## Refinement   

Crystal data, data collection and structure refinement details are summarized in Table 2 ▸. Hydrogen atoms of the amino group were located in a difference-Fourier map and refined freely. The hy­droxy H atom (H15) was included in the calculated position (AFIX 147; O-H = 0.84 Å) and refined with U_iso_(H) = 1.5*U*_eq_(O). All the C-bound H atoms were placed in calculated positions and refined using a riding model: C—H = 0.95–1.00 Å with *U*_iso_(H) = 1.2*U*_eq_(C).

As reported previously (*cf*. Cambridge Structural Database; Groom *et al.*, 2016 ▸) short H⋯H contacts (< 2.0 Å), involving the hy­droxy H atom and the methyl H atoms of the 3,5-di-*tert*-butyl-4-hy­droxy­phenyl moiety, were observed.

## Supplementary Material

Crystal structure: contains datablock(s) I, Global. DOI: 10.1107/S205698901901243X/eb2022sup1.cif

Structure factors: contains datablock(s) I. DOI: 10.1107/S205698901901243X/eb2022Isup2.hkl

CCDC reference: 1934997

Additional supporting information:  crystallographic information; 3D view; checkCIF report

## Figures and Tables

**Table 1:** Hydrogen-bond geometry (Å, °)

*D*—H⋯*A*	*D*—H	H⋯*A*	*D*⋯*A*	*D*—H⋯*A*
N28—H28*B*⋯N19	0.91 (2)	2.30 (2)	2.678 (3)	105 (2)
N28—H28*A*⋯O29^i^	0.88 (2)	2.03 (2)	2.912 (3)	174 (2)

Symmetry code: (i) 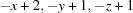.

**Table 2:** Experimental details

Crystal data	
Chemical formula	C_24_H_31_N_3_O_2_
*M*_r_	393.52
Crystal system, space group	Triclinic, *P* 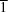
Temperature (K)	150
*a*, *b*, *c* (Å)	6.095 (3), 10.215 (4), 17.995 (8)
α, β, γ (°)	84.781 (15), 85.688 (15), 76.012 (15)
*V* (Å^3^)	1081.1 (8)
*Z*	2
Radiation type	Mo *K*α
μ (mm^−1^)	0.08
Crystal size (mm)	0.20 × 0.16 × 0.13

Data collection	
Diffractometer	Bruker APEXII CCD
Absorption correction	Multi-scan (*SADABS*; Bruker, 2003 ▸)
*T*_min_, *T*_max_	0.976, 0.982
No. of measured, independent and observed [*I* > 2σ(*I*)] reflections	27614, 5128, 3573
*R*_int_	0.093
(sin θ/λ)_max_ (Å^−1^)	0.659

Refinement	
*R*[*F* ^2^ > 2σ(*F* ^2^)], *wR*(*F* ^2^), *S*	0.055, 0.163, 1.03
No. of reflections	5128
No. of parameters	278
H-atom treatment	H atoms treated by a mixture of independent and constrained refinement
Δρ_max_, Δρ_min_ (e Å^−3^)	0.34, −0.20

Computer programs: *APEX2* and *SAINT* (Bruker, 2003 ▸), *SHELXT2014* (Sheldrick, 2015*a* ▸), *SHELXL2018/3* (Sheldrick, 2015*b* ▸), *ORTEP-3 for Windows* (Farrugia, 2012 ▸) and *PLATON* (Spek, 2009 ▸).

## supplementary crystallographic information

### Crystal data

**Table:**

C_24_H_31_N_3_O_2_	*Z* = 2
*M**_r_* = 393.52	*F*(000) = 424
Triclinic, *P*1	*D*_x_ = 1.209 Mg m^−^^3^
*a* = 6.095 (3) Å	Mo *K*α radiation, λ = 0.71073 Å
*b* = 10.215 (4) Å	Cell parameters from 7006 reflections
*c* = 17.995 (8) Å	θ = 2.3–27.5°
α = 84.781 (15)°	µ = 0.08 mm^−^^1^
β = 85.688 (15)°	*T* = 150 K
γ = 76.012 (15)°	Plate, colorless
*V* = 1081.1 (8) Å^3^	0.20 × 0.16 × 0.13 mm

### Data collection

**Table:**

Bruker APEXII CCD diffractometer	3573 reflections with *I* > 2σ(*I*)
φ and ω scans	*R*_int_ = 0.093
Absorption correction: multi-scan (SADABS; Bruker, 2003)	θ_max_ = 28.0°, θ_min_ = 2.3°
*T*_min_ = 0.976, *T*_max_ = 0.982	*h* = −8→8
27614 measured reflections	*k* = −13→13
5128 independent reflections	*l* = −23→23

### Refinement

**Table:**

Refinement on *F*^2^	Secondary atom site location: difference Fourier map
Least-squares matrix: full	Hydrogen site location: mixed
*R*[*F*^2^ > 2σ(*F*^2^)] = 0.055	H atoms treated by a mixture of independent and constrained refinement
*wR*(*F*^2^) = 0.163	*w* = 1/[σ^2^(*F*_o_^2^) + (0.0884*P*)^2^ + 0.1168*P*] where *P* = (*F*_o_^2^ + 2*F*_c_^2^)/3
*S* = 1.02	(Δ/σ)_max_ < 0.001
5128 reflections	Δρ_max_ = 0.34 e Å^−^^3^
278 parameters	Δρ_min_ = −0.20 e Å^−^^3^
0 restraints	Extinction correction: (SHELXL-2018/3; Sheldrick, 2015b), Fc^*^=kFc[1+0.001xFc^2^λ^3^/sin(2θ)]^-1/4^
Primary atom site location: dual	Extinction coefficient: 0.045 (10)

### Special details

**Table:**

Geometry. All esds (except the esd in the dihedral angle between two l.s. planes) are estimated using the full covariance matrix. The cell esds are taken into account individually in the estimation of esds in distances, angles and torsion angles; correlations between esds in cell parameters are only used when they are defined by crystal symmetry. An approximate (isotropic) treatment of cell esds is used for estimating esds involving l.s. planes.

### Fractional atomic coordinates and isotropic or equivalent isotropic displacement parameters (Å^2^)

**Table:**

	*x*	*y*	*z*	*U*_iso_*/*U*_eq_	
O15	0.9461 (2)	0.44716 (13)	0.91734 (7)	0.0566 (4)	
H15	1.033732	0.369748	0.919753	0.085*	
O29	0.8119 (2)	0.43979 (12)	0.57125 (7)	0.0481 (3)	
N19	0.3611 (2)	0.72589 (13)	0.58893 (7)	0.0346 (3)	
N20	0.5033 (2)	0.59925 (13)	0.60307 (7)	0.0370 (3)	
N28	0.7453 (3)	0.64582 (18)	0.50555 (9)	0.0523 (4)	
H28A	0.882 (4)	0.625 (2)	0.4835 (13)	0.067 (7)*	
H28B	0.659 (4)	0.732 (2)	0.5038 (12)	0.063 (6)*	
C1	0.8395 (2)	0.35284 (15)	0.81126 (8)	0.0317 (3)	
C2	0.8169 (3)	0.46205 (15)	0.85614 (8)	0.0344 (3)	
C3	0.6665 (2)	0.58842 (15)	0.84134 (8)	0.0314 (3)	
C4	0.5350 (2)	0.60223 (15)	0.77960 (8)	0.0313 (3)	
H4	0.429397	0.685704	0.768849	0.038*	
C5	0.5536 (2)	0.49774 (14)	0.73326 (8)	0.0293 (3)	
C6	0.7058 (2)	0.37542 (15)	0.74962 (8)	0.0314 (3)	
H6	0.719460	0.304401	0.717703	0.038*	
C7	1.0061 (3)	0.21512 (15)	0.82845 (9)	0.0355 (4)	
C8	0.9502 (4)	0.1510 (2)	0.90606 (11)	0.0568 (5)	
H8A	0.795804	0.137693	0.908130	0.085*	
H8B	0.961692	0.210912	0.944383	0.085*	
H8C	1.057342	0.063409	0.915125	0.085*	
C9	0.9923 (4)	0.11345 (18)	0.77254 (12)	0.0586 (6)	
H9A	0.838694	0.098943	0.775293	0.088*	
H9B	1.100388	0.027416	0.784629	0.088*	
H9C	1.029174	0.148899	0.721901	0.088*	
C10	1.2512 (3)	0.2323 (2)	0.82225 (13)	0.0581 (5)	
H10A	1.354990	0.145275	0.835704	0.087*	
H10B	1.265658	0.298983	0.856270	0.087*	
H10C	1.288924	0.263391	0.770813	0.087*	
C11	0.6506 (3)	0.70679 (16)	0.89082 (9)	0.0368 (4)	
C12	0.8800 (3)	0.7458 (2)	0.88750 (12)	0.0533 (5)	
H12A	0.924088	0.769747	0.835484	0.080*	
H12B	0.995181	0.669096	0.907726	0.080*	
H12C	0.866678	0.823474	0.917172	0.080*	
C13	0.4760 (3)	0.83347 (17)	0.86389 (11)	0.0509 (5)	
H13A	0.519650	0.862799	0.812705	0.076*	
H13B	0.469798	0.905832	0.896767	0.076*	
H13C	0.326834	0.812938	0.864961	0.076*	
C14	0.5774 (4)	0.6689 (2)	0.97178 (10)	0.0533 (5)	
H14A	0.569824	0.744823	1.002283	0.080*	
H14B	0.687629	0.589255	0.991622	0.080*	
H14C	0.427997	0.648596	0.973155	0.080*	
C16	0.4155 (2)	0.51573 (15)	0.66436 (8)	0.0318 (3)	
H16	0.417377	0.425207	0.646982	0.038*	
C17	0.1694 (3)	0.59885 (16)	0.67369 (9)	0.0358 (4)	
H17A	0.062980	0.553619	0.653335	0.043*	
H17B	0.124746	0.613620	0.726873	0.043*	
C18	0.1762 (3)	0.73077 (15)	0.62880 (8)	0.0318 (3)	
C21	−0.0090 (3)	0.85374 (15)	0.62682 (8)	0.0339 (3)	
C22	−0.2214 (3)	0.84869 (19)	0.65965 (10)	0.0443 (4)	
H22	−0.244531	0.766713	0.684641	0.053*	
C23	−0.3989 (3)	0.9624 (2)	0.65606 (11)	0.0540 (5)	
H23	−0.543486	0.957730	0.678261	0.065*	
C24	−0.3679 (4)	1.0824 (2)	0.62059 (11)	0.0559 (5)	
H24	−0.490868	1.159998	0.618221	0.067*	
C25	−0.1580 (4)	1.08977 (18)	0.58852 (10)	0.0526 (5)	
H25	−0.136101	1.172570	0.564262	0.063*	
C26	0.0211 (3)	0.97629 (17)	0.59169 (9)	0.0421 (4)	
H26	0.165500	0.981990	0.569754	0.050*	
C27	0.6958 (3)	0.55571 (17)	0.55953 (8)	0.0364 (4)	

### Atomic displacement parameters (Å^2^)

**Table:**

	*U*^11^	*U*^22^	*U*^33^	*U*^12^	*U*^13^	*U*^23^
O15	0.0598 (8)	0.0483 (8)	0.0558 (8)	0.0115 (6)	−0.0335 (7)	−0.0142 (6)
O29	0.0416 (7)	0.0390 (7)	0.0552 (7)	0.0044 (5)	0.0052 (5)	−0.0021 (5)
N19	0.0345 (7)	0.0325 (7)	0.0321 (6)	0.0012 (5)	−0.0051 (5)	0.0004 (5)
N20	0.0347 (7)	0.0341 (7)	0.0346 (7)	0.0038 (6)	−0.0014 (5)	0.0040 (5)
N28	0.0468 (9)	0.0471 (9)	0.0509 (9)	0.0039 (7)	0.0122 (7)	0.0060 (7)
C1	0.0278 (7)	0.0277 (7)	0.0364 (8)	0.0002 (6)	−0.0051 (6)	−0.0013 (6)
C2	0.0324 (8)	0.0331 (8)	0.0357 (8)	−0.0012 (6)	−0.0106 (6)	−0.0023 (6)
C3	0.0300 (7)	0.0275 (7)	0.0356 (7)	−0.0042 (6)	−0.0035 (6)	−0.0029 (6)
C4	0.0288 (7)	0.0253 (7)	0.0371 (7)	−0.0013 (6)	−0.0056 (6)	0.0009 (5)
C5	0.0271 (7)	0.0280 (7)	0.0316 (7)	−0.0045 (6)	−0.0037 (6)	0.0006 (5)
C6	0.0307 (7)	0.0279 (7)	0.0342 (7)	−0.0031 (6)	−0.0043 (6)	−0.0036 (6)
C7	0.0319 (8)	0.0293 (8)	0.0403 (8)	0.0038 (6)	−0.0082 (6)	−0.0012 (6)
C8	0.0649 (13)	0.0419 (10)	0.0526 (11)	0.0039 (9)	−0.0012 (9)	0.0082 (8)
C9	0.0647 (12)	0.0359 (9)	0.0659 (12)	0.0164 (9)	−0.0251 (10)	−0.0144 (8)
C10	0.0343 (9)	0.0522 (11)	0.0808 (14)	0.0033 (8)	−0.0065 (9)	−0.0010 (10)
C11	0.0372 (8)	0.0305 (8)	0.0420 (8)	−0.0035 (6)	−0.0070 (7)	−0.0073 (6)
C12	0.0489 (11)	0.0490 (11)	0.0676 (12)	−0.0172 (9)	−0.0082 (9)	−0.0148 (9)
C13	0.0564 (11)	0.0323 (9)	0.0596 (11)	0.0044 (8)	−0.0117 (9)	−0.0130 (8)
C14	0.0617 (12)	0.0520 (11)	0.0436 (10)	−0.0063 (10)	0.0000 (9)	−0.0113 (8)
C16	0.0302 (7)	0.0288 (7)	0.0350 (7)	−0.0033 (6)	−0.0073 (6)	0.0002 (6)
C17	0.0290 (8)	0.0358 (8)	0.0409 (8)	−0.0043 (6)	−0.0092 (6)	0.0025 (6)
C18	0.0311 (7)	0.0335 (8)	0.0296 (7)	−0.0028 (6)	−0.0078 (6)	−0.0028 (6)
C21	0.0353 (8)	0.0339 (8)	0.0298 (7)	−0.0003 (6)	−0.0085 (6)	−0.0045 (6)
C22	0.0351 (9)	0.0435 (10)	0.0512 (10)	−0.0024 (7)	−0.0059 (7)	−0.0036 (7)
C23	0.0362 (9)	0.0607 (12)	0.0585 (11)	0.0057 (8)	−0.0069 (8)	−0.0123 (9)
C24	0.0552 (12)	0.0488 (11)	0.0515 (11)	0.0191 (9)	−0.0180 (9)	−0.0130 (8)
C25	0.0709 (13)	0.0349 (9)	0.0442 (9)	0.0049 (9)	−0.0136 (9)	−0.0006 (7)
C26	0.0491 (10)	0.0379 (9)	0.0354 (8)	−0.0022 (7)	−0.0059 (7)	−0.0016 (6)
C27	0.0326 (8)	0.0388 (8)	0.0345 (8)	−0.0009 (7)	−0.0029 (6)	−0.0048 (6)

### Geometric parameters (Å, º)

**Table:**

O15—C2	1.3776 (19)	C10—H10B	0.9800
O15—H15	0.8400	C10—H10C	0.9800
O29—C27	1.233 (2)	C11—C13	1.529 (2)
N19—C18	1.282 (2)	C11—C14	1.534 (3)
N19—N20	1.3859 (18)	C11—C12	1.539 (3)
N20—C27	1.364 (2)	C12—H12A	0.9800
N20—C16	1.480 (2)	C12—H12B	0.9800
N28—C27	1.345 (2)	C12—H12C	0.9800
N28—H28A	0.88 (3)	C13—H13A	0.9800
N28—H28B	0.91 (2)	C13—H13B	0.9800
C1—C6	1.395 (2)	C13—H13C	0.9800
C1—C2	1.410 (2)	C14—H14A	0.9800
C1—C7	1.544 (2)	C14—H14B	0.9800
C2—C3	1.407 (2)	C14—H14C	0.9800
C3—C4	1.395 (2)	C16—C17	1.540 (2)
C3—C11	1.546 (2)	C16—H16	1.0000
C4—C5	1.392 (2)	C17—C18	1.515 (2)
C4—H4	0.9500	C17—H17A	0.9900
C5—C6	1.386 (2)	C17—H17B	0.9900
C5—C16	1.523 (2)	C18—C21	1.472 (2)
C6—H6	0.9500	C21—C22	1.393 (2)
C7—C9	1.531 (2)	C21—C26	1.397 (2)
C7—C8	1.538 (3)	C22—C23	1.383 (2)
C7—C10	1.541 (3)	C22—H22	0.9500
C8—H8A	0.9800	C23—C24	1.377 (3)
C8—H8B	0.9800	C23—H23	0.9500
C8—H8C	0.9800	C24—C25	1.380 (3)
C9—H9A	0.9800	C24—H24	0.9500
C9—H9B	0.9800	C25—C26	1.387 (2)
C9—H9C	0.9800	C25—H25	0.9500
C10—H10A	0.9800	C26—H26	0.9500
			
C2—O15—H15	109.5	C12—C11—C3	109.96 (13)
C18—N19—N20	108.15 (13)	C11—C12—H12A	109.5
C27—N20—N19	121.43 (13)	C11—C12—H12B	109.5
C27—N20—C16	124.77 (13)	H12A—C12—H12B	109.5
N19—N20—C16	113.61 (12)	C11—C12—H12C	109.5
C27—N28—H28A	116.5 (15)	H12A—C12—H12C	109.5
C27—N28—H28B	118.7 (14)	H12B—C12—H12C	109.5
H28A—N28—H28B	122 (2)	C11—C13—H13A	109.5
C6—C1—C2	116.81 (13)	C11—C13—H13B	109.5
C6—C1—C7	121.23 (13)	H13A—C13—H13B	109.5
C2—C1—C7	121.95 (13)	C11—C13—H13C	109.5
O15—C2—C3	117.36 (13)	H13A—C13—H13C	109.5
O15—C2—C1	119.80 (13)	H13B—C13—H13C	109.5
C3—C2—C1	122.84 (13)	C11—C14—H14A	109.5
C4—C3—C2	116.96 (13)	C11—C14—H14B	109.5
C4—C3—C11	121.73 (13)	H14A—C14—H14B	109.5
C2—C3—C11	121.31 (13)	C11—C14—H14C	109.5
C5—C4—C3	122.16 (13)	H14A—C14—H14C	109.5
C5—C4—H4	118.9	H14B—C14—H14C	109.5
C3—C4—H4	118.9	N20—C16—C5	111.58 (12)
C6—C5—C4	118.82 (13)	N20—C16—C17	100.20 (11)
C6—C5—C16	119.48 (13)	C5—C16—C17	115.44 (13)
C4—C5—C16	121.69 (13)	N20—C16—H16	109.7
C5—C6—C1	122.39 (14)	C5—C16—H16	109.7
C5—C6—H6	118.8	C17—C16—H16	109.7
C1—C6—H6	118.8	C18—C17—C16	102.79 (13)
C9—C7—C8	106.08 (16)	C18—C17—H17A	111.2
C9—C7—C10	107.04 (16)	C16—C17—H17A	111.2
C8—C7—C10	110.82 (15)	C18—C17—H17B	111.2
C9—C7—C1	111.32 (13)	C16—C17—H17B	111.2
C8—C7—C1	111.35 (13)	H17A—C17—H17B	109.1
C10—C7—C1	110.08 (14)	N19—C18—C21	121.19 (14)
C7—C8—H8A	109.5	N19—C18—C17	113.38 (13)
C7—C8—H8B	109.5	C21—C18—C17	125.40 (14)
H8A—C8—H8B	109.5	C22—C21—C26	118.56 (15)
C7—C8—H8C	109.5	C22—C21—C18	119.78 (15)
H8A—C8—H8C	109.5	C26—C21—C18	121.66 (15)
H8B—C8—H8C	109.5	C23—C22—C21	120.35 (18)
C7—C9—H9A	109.5	C23—C22—H22	119.8
C7—C9—H9B	109.5	C21—C22—H22	119.8
H9A—C9—H9B	109.5	C24—C23—C22	120.60 (19)
C7—C9—H9C	109.5	C24—C23—H23	119.7
H9A—C9—H9C	109.5	C22—C23—H23	119.7
H9B—C9—H9C	109.5	C23—C24—C25	119.85 (17)
C7—C10—H10A	109.5	C23—C24—H24	120.1
C7—C10—H10B	109.5	C25—C24—H24	120.1
H10A—C10—H10B	109.5	C24—C25—C26	120.03 (18)
C7—C10—H10C	109.5	C24—C25—H25	120.0
H10A—C10—H10C	109.5	C26—C25—H25	120.0
H10B—C10—H10C	109.5	C25—C26—C21	120.60 (18)
C13—C11—C14	106.92 (15)	C25—C26—H26	119.7
C13—C11—C12	106.76 (15)	C21—C26—H26	119.7
C14—C11—C12	110.53 (15)	O29—C27—N28	124.31 (15)
C13—C11—C3	111.83 (13)	O29—C27—N20	119.69 (15)
C14—C11—C3	110.74 (14)	N28—C27—N20	116.00 (15)
			
C18—N19—N20—C27	168.45 (14)	C27—N20—C16—C5	74.87 (19)
C18—N19—N20—C16	−6.82 (17)	N19—N20—C16—C5	−110.05 (14)
C6—C1—C2—O15	−179.18 (15)	C27—N20—C16—C17	−162.42 (14)
C7—C1—C2—O15	0.0 (2)	N19—N20—C16—C17	12.66 (16)
C6—C1—C2—C3	0.6 (2)	C6—C5—C16—N20	−104.30 (16)
C7—C1—C2—C3	179.85 (14)	C4—C5—C16—N20	74.21 (18)
O15—C2—C3—C4	−179.49 (14)	C6—C5—C16—C17	142.19 (14)
C1—C2—C3—C4	0.7 (2)	C4—C5—C16—C17	−39.3 (2)
O15—C2—C3—C11	1.3 (2)	N20—C16—C17—C18	−12.66 (14)
C1—C2—C3—C11	−178.56 (15)	C5—C16—C17—C18	107.30 (14)
C2—C3—C4—C5	−1.4 (2)	N20—N19—C18—C21	179.27 (12)
C11—C3—C4—C5	177.80 (14)	N20—N19—C18—C17	−2.84 (17)
C3—C4—C5—C6	0.8 (2)	C16—C17—C18—N19	10.54 (16)
C3—C4—C5—C16	−177.68 (14)	C16—C17—C18—C21	−171.67 (13)
C4—C5—C6—C1	0.6 (2)	N19—C18—C21—C22	168.31 (14)
C16—C5—C6—C1	179.16 (14)	C17—C18—C21—C22	−9.3 (2)
C2—C1—C6—C5	−1.3 (2)	N19—C18—C21—C26	−10.7 (2)
C7—C1—C6—C5	179.48 (14)	C17—C18—C21—C26	171.63 (14)
C6—C1—C7—C9	−2.4 (2)	C26—C21—C22—C23	1.2 (2)
C2—C1—C7—C9	178.43 (16)	C18—C21—C22—C23	−177.89 (15)
C6—C1—C7—C8	−120.56 (17)	C21—C22—C23—C24	−0.5 (3)
C2—C1—C7—C8	60.3 (2)	C22—C23—C24—C25	−0.2 (3)
C6—C1—C7—C10	116.12 (17)	C23—C24—C25—C26	0.3 (3)
C2—C1—C7—C10	−63.0 (2)	C24—C25—C26—C21	0.3 (3)
C4—C3—C11—C13	0.8 (2)	C22—C21—C26—C25	−1.1 (2)
C2—C3—C11—C13	180.00 (15)	C18—C21—C26—C25	177.97 (14)
C4—C3—C11—C14	119.93 (17)	N19—N20—C27—O29	−176.12 (14)
C2—C3—C11—C14	−60.9 (2)	C16—N20—C27—O29	−1.4 (2)
C4—C3—C11—C12	−117.62 (17)	N19—N20—C27—N28	4.0 (2)
C2—C3—C11—C12	61.6 (2)	C16—N20—C27—N28	178.67 (15)

### Hydrogen-bond geometry (Å, º)

**Table:**

*D*—H···*A*	*D*—H	H···*A*	*D*···*A*	*D*—H···*A*
N28—H28*B*···N19	0.91 (2)	2.30 (2)	2.678 (3)	105 (2)
N28—H28*A*···O29^i^	0.88 (2)	2.03 (2)	2.912 (3)	174 (2)

Symmetry code: (i) −*x*+2, −*y*+1, −*z*+1.
